# Analysis of Physical–Cognitive Tasks Including Feedback-Based Technology for Alzheimer’s Disorder in a Randomized Experimental Pilot Study

**DOI:** 10.3390/jcm12175484

**Published:** 2023-08-24

**Authors:** Maria-Luisa Benitez-Lugo, Manuel Vazquez-Marrufo, Elena Pinero-Pinto, Gema Chamorro-Moriana, Veronica Perez-Cabezas, Carmen Suarez-Serrano

**Affiliations:** 1 Department of Physiotherapy, Faculty of Nursery, Physiotherapy and Podiatry, University of Seville, 41009 Seville, Spain; marisabeni@us.es (M.-L.B.-L.); csuarez@us.es (C.S.-S.); 2 Department of Experimental Phycology, Faculty of Phycology, University of Seville, 41018 Seville, Spain; 3 Department of Physiotherapy, Faculty of Nursery and Physiotherapy, University of Cadiz, 11009 Cadiz, Spain; veronica.perezcabezas@uca.es

**Keywords:** Alzheimer’s disease, balance, gait, cognitive decline, exercise, attention networks, feedback-based technology, prevention

## Abstract

Introduction: Alzheimer’s disease causes great changes, with the prefrontal cortex being the most frequently damaged zone; these changes affect physical and cognitive behavior and compromise autonomy. Objective: The objective of this study was to evaluate the effects of physical–cognitive tasks on memory, attention, balance, gait, and risk of falling in Alzheimer’s by using feedback-based technology. Methods: Forty patients with Alzheimer’s were recruited from an Alzheimer’s Association; of these, 15 met the inclusion criteria and were included in the pilot RCT (eight in the control group; seven in the experimental group). Assessment tools: The Cognitive Mini-Examination Scale, Oddball Test and Attention Network, Berg Scale, Tinetti, Timed Up and Go, and Geriatric Deterioration Scale. The experimental group was treated with physical–cognitive tasks by using combined feedback-based technology (visual, acoustic, simultaneous, immediate, and terminal feedback, as well as knowledge of the results and performance) under the supervision of physiotherapists twice per week for 16 thirty-minute sessions. The control group underwent their usual care (pharmacological treatment, mobility exercises, and cognitive stimulation sessions). Result: In the experimental group, the contrast tests showed differences for the re-test (except in attention), with the significative Timed Up and Go test being significant (*p* = 0.020). The interaction between groups showed significant differences for the experimental group according to the MEC (*p* = 0.029; *d* = 0.14) and Tinetti (*p* = 0.029; *d* = 0.68). Discussion/Conclusion: Memory, balance, gait, and risk of falling improved in the Alzheimer’s patients through the use of physical–cognitive tasks involving combined feedback-based technology. The effects on attention were inconclusive. The outcomes should be treated with caution due to the sample. This can promote intergenerational bonds, use at home, and adherence to treatment.

## 1. Introduction

The aging rate of Western countries, including Spain, continues to grow exponentially [1,2]. The aging process, especially in Alzheimer’s disease, causes a decline at the level of different organs and systems, highlighting the prefrontal cortex as one of the areas that is most affected by the passage of time. This structure is crucial for executive skills, attentional abilities, and the capacity to be resilient [3]. Executive functions and gait are closely connected [4], and so, the impairment of either has cognitive and physical repercussions. Specifically, the relation between attentional capacity and Alzheimer’s disease has been shown [5], as has the role of the executive and frontoparietal networks [6]. There are also findings about the associations between physical frailty, cognitive impairment, and risk of falling [7,8]. Although it has been less studied, the cognitive function of attention is affected by this disease and, thus, is often responsible for the first memory problems in patients. Understanding the anatomical and functional basis of Alzheimer’s is one of the great challenges of neuroscience [9]. The three attentional networks (alert, orientation, and executive) described by Posner et al. [9] are globally affected due to frontostriatal and frontoparietal degeneration, a decrease in occipital volume, and the loss of gray and white matter [10]. These findings were analyzed with an attention network test (ANT) [9] and other functional tests. Previous data could explain the impairment in cognitive performance and, consequently, the necessity of treatments that stimulate attentional networks [10].

Therefore, Alzheimer’s disorders imply brain changes that lead to physical, cognitive, emotional, and social manifestations [11]. Interventional therapies are currently applied because there is no cure for this disorder. Knowing the risk factors (lack of movement, stress, nutrition, etc.) can help clinicians make decisions on therapies. [12]. Physical activities improve brain functions and delay Alzheimer’s by activating brain vascularization, plasticity, and neurogenesis and by reducing inflammation due to a decrease in the production of amyloid-beta peptides (Aβ) [13]. Consequently, cognitive functions would also improve [13]. In addition, combinations of cognitive- and physical-challenge-oriented tasks (dual tasks) were found to optimize the results of training in Alzheimer’s [14,15,16].

Informational technology (IT) allows patients to develop motor skills, e.g., postural control and gait, and cognitive skills. In addition, IT is easily combined with feedback-based treatments by using electronic devices in the clinical field. Extrinsic feedback provides the possibility of voluntarily controlling and changing bodily functions and/or biological processes through the information that the subject receives from it, as it makes subjects aware of situations that they do not voluntarily appreciate. These therapies usually incorporate inertial monitoring devices, robot-assisted movement, or virtual reality technology, and they can use visual, acoustic, or haptic feedback systems [17]. In addition, other types of feedback could be considered based on the therapies’ objectives: terminal/delayed feedback, simultaneous/immediate feedback, descriptive feedback, prescriptive feedback, knowledge of the result (KR), and knowledge of the performance (KP), among others. If therapy is applied through video games, the results could improve because, in addition to their working movement and three attentional networks [10], patients enjoy themselves, and the social interactions between generations are increased [18]. Previous studies have already shown the benefits of video game use for balance and risk of falling [19,20,21].

The methods described above are effective for gait assessment and reeducation [17], which are issues that are especially relevant in Alzheimer’s, since gait disturbances may be associated with a decline in cognitive function and may even indicate the first signs of this disorder [22].

The background of attentional networks and the benefits found for cognition, gait, balance, and avoidance of falls in the elderly and Alzheimer’s patients through the application of feedback-based technology led us to consider further research on this issue. Hence, this study aimed to evaluate the effects of physical–cognitive tasks involving feedback-based technology on memory, attention, balance, gait, and risk of falling in Alzheimer’s patients.

## 2. Materials and Methods

A randomized pilot clinical trial based on a previous study [23] was planned to assess the efficacy of the use of a technological tool with feedback (Nintendo^®^ Wii, Video Console) on the physical–cognitive capacities of older subjects with Alzheimer’s disorder. This research was based on the Declaration of Helsinki [24] and approved by an experimental ethics committee (University of Seville). The legal guardians of the patients signed informed consent forms. The subjects were randomized by using sealed envelopes, and parallel groups were formed: control and experimental groups. The protocol followed the recommendations provided in the SPIRIT [25] statement.

This clinical trial was registered in Clinical Trials Gov (2020) with the code NCT04615897.

Likewise, the Oxford [26] and Consort [27] standards were considered for the development of the trial. The patients and the professionals who performed the assessments were blinded. Physiotherapists who delivered the therapy with the feedback-based technology were not blinded.

### 2.1. Participants 

The patients were referred from the *Santa Elena Alzheimer Association* in Seville (Spain). The inclusion criteria were the following: Alzheimer’s diagnosed with the Diagnostic and Statistical Manual of Mental Disorders (DSM-IV criteria) by the patients’ neurologists, reaching a value on the “Geriatric Deterioration Scale” of ≤5 (cognitive stage range: no decline = 1; very mild = 2; mild = 3; severe = 4; moderate severe = 5; moderately severe = 6; very severe = 7), and walking without technical assistance. Patients excluded from the study were those with uncompensated hearing or visual disturbances and musculoskeletal limitations, those who had discontinued their therapy with the association (independent sessions of mobility exercises and cognitive stimulation), those who had discontinued regular drug usage, those who had hallucinations or delusional ideations (whether due to drugs or not), and those who had sleep habits of less than 6 h a day.

### 2.2. Variables

The variables considered in this study were moderating variables (sex, weight, age, dominance, level of education (basic studies—being able to read and write; primary education—schooling for 6–12 years; secondary education—schooling for 13–16 years), phase of the disease, hours of sleep, additional diseases, and medication), dependent variables by outcome measure (see below), and independent variables related to the intervention.

### 2.3. Outcome Measures

This study contained the following five dependent variables.

– Attention: This was assessed with the Oddball test (first) and ANT-Elderly (second) test [28]. The tasks of both tests required the patient to respond to an objective stimulus. In the Oddball test, the patient had to react by pressing a button before the appearance of red and white chessboards. This task involved 200 tries, of which 50 required a response. In the ANT-Elderly test, 5 arrows (imperative stimulus) pointing to the right or left were displayed. They did not necessarily have to point to the same side. The arrows had the following characteristics: a width of 7.37°, a height of 0.86°, and a duration of 350 ms. The patients had to click the right or left mouse button depending on the central arrow, which was presented as a target stimulus. As a previous stimulus, a cue (clue) could appear to indicate when the arrows would appear or where on the screen they would appear. The cues had the following characteristics (asterisk): a width of 0.86°, a height of 0.86°, and a duration of 150 ms. The time between the cue and the arrows was 850 ms, and the time between the arrows and the next cue ranged from 150 to 1000 ms. The appearance or non-appearance of cues was programmed into the tool to objectively assess the different attentional networks. The Attention Network test was separated into two parts (72 trials/a 5 min break to avoid fatigue/72 trials). The rest of the data on this tool can be found in [23].
– Memory: This was assessed with the Mini-Mental State Examination (MMSE). At the end of the test, the patients received a score that determined the presence or absence of a cognitive decline. The Spanish version, which is called the Cognitive Mini-Examination (MEC), consists of 30 items that are grouped into five parts: orientation, memorization, retrospective memory, concentration and calculation, and language and construction. The sum the parts leads to the total score. The cut-off point for dementia was set at 24. Values closer to 30 corresponded to optimal cognitive abilities.
– Balance: This outcome was measured with the application of the Berg scale [29]. The scale allowed the quantification of balance in different positions, resulting in a global score that guided the balance function in general, which could be used to predict the possibility of the use of a wheelchair, other technical aids, or total independence for the development of the gait. The patients executed 14 different items, with each of them being valued between 0 and 4 points. The total value was provided by the sum of the items. The interpretation of balance could be good (41–56 points), compromised (21–40 points), and affected (0–20 points).
– Gait: This was evaluated through the Timed Up and Go test [30]. This test measured how long it took for a subject to get up from a chair, walk three meters, and go back to the starting position. A patient’s gait was considered normal gait if they took less than 10 s to perform the test; they were considered to have a slight risk of falling if the test time was between 11 and 20 s and a high risk of falling if it took more than 20 s to perform the test.
– Functionality and risk of falls [31]: This outcome was measured through the application of the Tinetti scale, an instrument that evaluates balance, gait, and falls. There were 16 items in total, and the total score was 28 points. The scale had two dimensions: balance (9 items), with a score between 0 and 16, and gait (7 items), with a score between 0 and 12 points. The interpretation of the functionality and risk of falls was as follows: high risk (<19 points); risk (19–23 points); low or slight risk (24–28 points).

### 2.4. Treatment Using Feedback-Based Technology

A Nintendo^®^ Wii with a long remote and gyroscopic technology was used. Movements, distances, angles, and speeds were detected with a wireless device. This device offered acoustic and visual feedback. The main technical data were as follows: an IBM Broadway 729 MHz processor, a 512 MB flash memory card, SD and SDHC cards, a Nintendo GameCube memory card, a graphics ATI Hollywood 243 MHz, Wi-Fi, Bluetooth 2.0, 2 × USB 2.0, and a LAN adaptor via USB 2.0.

The tools had different types of feedback, with all of them being extrinsic: acoustic and visual feedback, terminal/delayed feedback, simultaneous/immediate feedback, descriptive feedback, prescriptive feedback, knowledge of the result (KR), and knowledge of the performance (KP), among others. The various types of feedback that were applied in each game could change.

**Figure 1:**
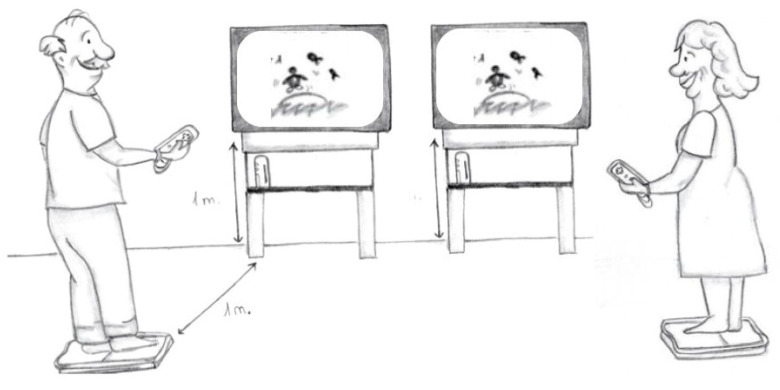
Task performance: device locations and patients.

The games (Wii-Fit©) required the “Wii Balance Board”, a platform with four sensors that detected the pressure exerted on it. The sensors were associated with the wireless controller and with a display by using a movement bar. Later, each user was registered, and their specific characteristics were evaluated. Four exercise modalities were possible: yoga, toning, balance, and aerobics. This research used the following games: “Penguin Slide” for training balance and “Step Plus” for training balance and aerobic exercises. The distance between the “Wii balance board” and the screen was one meter, and the screen was placed on a one-meter-high desk (Figure 1). “Penguin Slide” required the transfer of the load to both sides, the variation of the center of gravity, and the concentration of the users throughout the task in order to collect fish that jumped. The patients obtained points and feedback both visually and acoustically while collecting fish. “Step Plus” consisted of stepping on and off the platform according to the rhythm of steps, thus simulating gait and training balance (static, reactive, and proactive). The patients obtained feedback again.

Feedback used in Penguin Slide: The balance was reported with simultaneous visual feedback (without a fall); with terminal, visual, and acoustic feedback (with a fall); or KP and visual feedback (at the end of the game with the scores). The fishing was reported with immediate feedback, which was visual and acoustic (after catching a fish), or KR (with the scores at the end of the game).

Feedback used in Step Plus: The performance was reported with immediate visual and acoustic feedback (after each step that touched the platform), with KR (with scores) and KP (with data on the time taken), and with visual feedback with colors and acoustic feedback (at the end of the game).

### 2.5. Laboratory Conditions

Three separate rooms were used. Two of them, in which a therapist and a single patient, accompanied by their main caregiver, were located, were used for the assessments. Each assessment room was 20 m^2^ and was equipped with a scale; a stretcher; a table; three chairs for the patient, companion, and assessor; a PC to perform the attention tests; scales/questionnaires/paper tests/informed consent forms (one per user); a stopwatch to measure the Timed Up and Go test and a Berg Scale item; a tape measure to calculate the reach distances on the Berg Scale and gait paths; a watch; and paper and a pen for the cognitive test.

The third room, which was intended for interventions, was 26 m^2^ in area and contained equipment for the treatment of two patients at the same time: two video consoles, two 32″ monitors placed on one-meter-high desks, and 2 platforms (see Figure 1). In this room, there were two therapists, one for each patient. They had chairs, tables, and record sheets for each of them, where they wrote down the scores achieved in each game.

All rooms were soundproofed and had windows, ventilation, and artificial light to provide optimal laboratory conditions.

The following clothing was requested from the patients: comfortable clothing and closed shoes with non-slip soles (i.e., sports shoes). Patients who wore glasses or hearing aids were asked to bring them with them.

### 2.6. Intervention Protocol and Phases of the Research

Table 1 shows the timeline developed in the research for each patient.

The research was divided into seven phases.

– Phase I: On the first day, the legal guardians read and signed the informed consent form. Subsequently, the users, in the presence of their companions, were brought together for a preliminary assessment and anamnesis that included questions on moderating variables (e.g., gender, dominance, drugs, etc.). Three days later, the patients who met the inclusion criteria were assessed with computerized tests and functional assessment scales/tests to determine their physical–cognitive capacities.
– Phase II: This phase involved the collection of the initial assessments by each evaluator and, later, the insertion of these data into a database (i.e., ratings of each of the two observers and the average of the two).
– Phase III: This phase involved treatment by using technology (games) in the intervention group. The total number of 30 min sessions was 16, with a frequency of 2 times per week [32]. Each session consisted of 3 series of “Penguin Slide”, 1 series of “Step Plus”, and 3 series more of “Penguin Slide”. The scores obtained in the sessions were recorded on a sheet to encourage the patients and the caregivers. The infrastructure of this study made it possible to test two users at the same to improve their motivation and adherence. The control group continued with their conventional treatment (independent sessions of mobility exercises and cognitive stimulation in their Alzheimer’s association).
– Phase IV: This phase involved the appointment of the patients for the final assessment and performance of the tests so that the physiotherapists and neuropsychologists could determine their physical–cognitive capacities.
– Phase V: This phase involved the total assessments of each observer and the average scores, as well as the insertion of the data into the database.
– Phase VI: In this phase, the database was completed, the data were blinded to the observers, and statistical analyses were performed.
– Phase VII: Once the data collection was finished, the control group received the same treatment as the intervention group.

### 2.7. Data Collection Procedures

The physiotherapists and neuropsychologists assessing the patients had more than ten years of experience in this area. The evaluations were carried out two times for both groups: before the intervention and seventy-two hours after the last session of the experimental group.

The E-prime program and statistical package in SPSS 18.0 were used.

The statistical analysis was as follows.

(1) The Shapiro–Wilk statistic was employed for normality tests on the dependent variables. The mean and standard deviation or median and interquartile range (based on the normality of the variables) were used to report the results of the dependent variables.

(2) The homogeneity of both groups was verified before starting the treatment. In the case of variables that were adjusted to the normal, we used Student’s *t*-test for independent samples (or Welch’s *t*-test when there was no homogeneity of the variances), and for those that did not adjust to the normal distribution, the Mann–Whitney U was used. Fisher’s exact test was performed for the gender variable.

(3) We proceeded to evaluate the existence of significant differences between tests and re-tests in each of the groups. Student’s t for related samples was used for the parametric variables, and the Wilcoxon ranks were used for the non-parametric variables.

(4) Lastly, the means of the different variables on the MEC scale, Berg Scale, Tinetti, Timed Up and Go test, Oddball test, and ANT-Elderly test were calculated to compare the two groups. Student’s t for independent samples or the Mann–Whitney U were applied in same way.

(5) The effect size was calculated for the parametric variables (d = 2t/√gl). The Grissom criteria were applied for the non-parametric variables. A 95% confidence interval (*p*-value < 0.05) was considered in the statistical analysis.

### 2.8. Ethical Considerations of the Study

The Research Ethics Committee of the University of Seville (Spain) authorized this study. The patients and the caregivers were informed about the research. Informed consent was obtained from all of the participants’ legal guardians before enrollment in the study. The protocols followed the Declaration of Helsinki. According to Law 15/1999 on the Protection of Personal Data, the personal data that we required from the subjects were those necessary to carry out the study correctly. The investigators involved in the study did not disclose any participants’ information to anyone outside of the research team. Participation was anonymous, but the data of all the participants were listed and protected by the leading investigator, who referred to them at crucial moments.

## 3. Results

**Figure 2:**
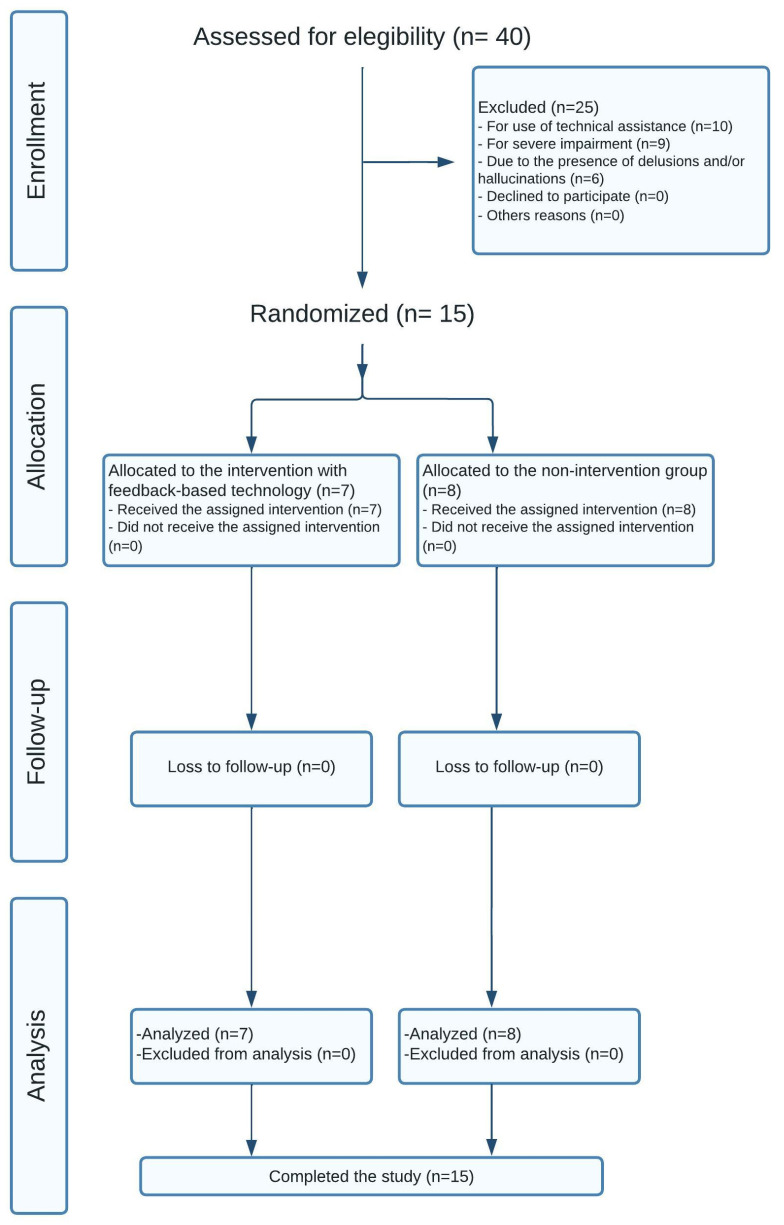
Flowchart of the study’s protocol.

After carrying out all of the research phases presented in the flowchart (Figure 2), the final sample consisted of 15 patients: eight subjects in the control group with a mean age of 79 (men: 37.5%; women: 62.5%) and seven subjects in the experimental group with a mean age of 79 (men: 14.3%; women: 85.7%).

The characteristics of the patients (group, socio-demographic characteristics, anthropometric characteristics, clinical characteristics, etc.) from the initial anamnesis are shown in Table 2.

The distribution between the control group and experimental group was not statistically significantly different in terms of the genders of the patients (*p* (bilateral) = 0.569). In the homogeneity tests, the analysis showed similar conditions for the respective variables for each group (Table 3).

The results found in the clinical trial in relation to the variables that were analyzed are shown in Table 4, which compares both the differences between the tests and re-tests in each of the groups as well as the differences between the groups. The statistically significant differences are highlighted.

**Figure 3:**
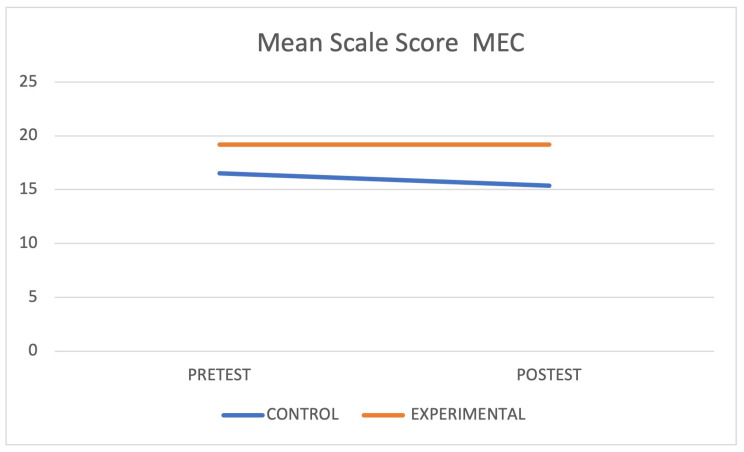
Mean scores on the MEC scale.

**Figure 4:**
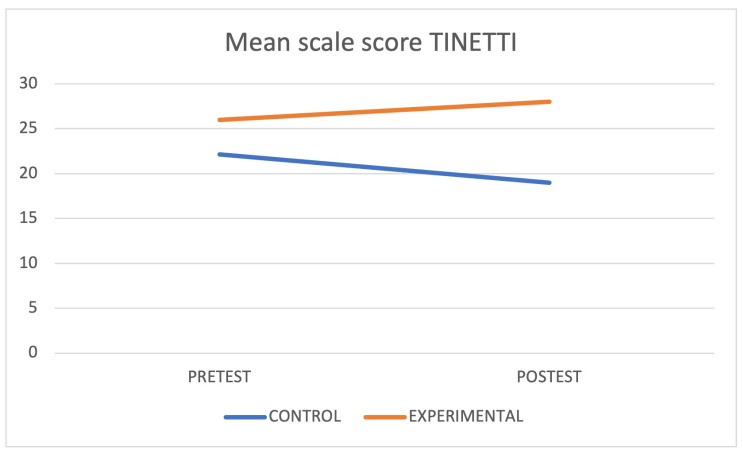
Mean scores on the Tinetti scale.

**Figure 5:**
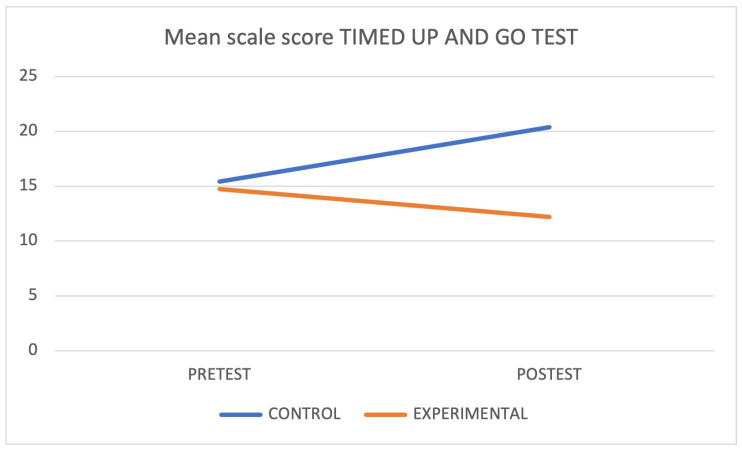
Mean scores on the Timed Up and Go test.

When each group was isolated and compared, a statistically significant difference was found in the Cognitive Mini-Examination (*p* < 0.05) and Tinetti Scale (*p* < 0.05) in the control group and in the Timed Up and Go (*p* < 0.05) in the intervention group. Figure 3, Figure 4 and Figure 5 graphically express these differences through the marginal means. Finally, the interaction between the groups showed statistically significant differences (*p* < 0.05) in the Cognitive Mini-Examination with a small effect size (*d* = 0.14), the Tinetti Scale with a moderate effect size (*d* = 0.68), and the Executive Network with a large effect size (*d* = 1.19).

## 4. Discussion

This research evaluated the effects of using feedback-based technology for physical–cognitive tasks on memory, attention, balance, gait, and risk of falling in Alzheimer’s patients; improvements were found in all of them apart from attention, i.e., the effect on the Executive Network decreased.

Regarding physical capacities, the motor skill exercises with feedback seemed to be effective for improving balance and gait (with a difference in the Tinetti variable; *p* = 0.029; *d* = 0.68). The other variables that quantified balance and gait (Berg scale; Timed Up and Go Test) did not show significant differences, although the descriptive analysis pointed to an improvement in the results of the physical measurements applied in the experimental group with respect to the control group. Even so, the absence of worsening is considered a therapeutic achievement, as Alzheimer’s is a degenerative disease. These findings coincide with those of Padala et al. [33]. It would be necessary to increase the sample size and design multicenter studies in order to achieve more robust results. Agmon et al. [34] also achieved similar results regarding physical abilities in Alzheimer’s patients when involving IT. With regard to cognitive abilities, the motor skill tasks seemed to be effective in the global improvement of cognition (with a difference in the MEC variable; *p* = 0.029; *d* = 0.14). This finding coincides with that of Maci et al. [35], who determined the effects of cognitive activities, physical activities, and socialization on the course of the disease, although the main objective of their study focused on caregivers. Tools that mix cognitive, physical, and social aspects seem to be effective for these patients [15,16]. The findings of the present trial supported this idea, thus advocating for this type of activity for improving the quality of life in patients and minimizing the burnout syndrome in their caregivers.

On the other hand, according to Yamaguchi et al. [36], when assessed with the MEC, memory improved after an intervention, although the effect on the executive network decreased in the intervention group with respect to the control. The results showed only a unilaterally significant difference in the executive variable (*p* = 0.04; *d* = 1.19). The justification for this could be the difficulty of the instrument for our sample, which showed lower accuracy than that of others [37,38], as well as the complex circuits that hide attentional networks. In addition, global tests such as the MEC evaluate the brain as a whole, but they do not do so specifically. The authors believe that if specific neural circuits were analyzed, the results could be different, and even more so when assessing damaged brains. The routine designed in the ANT had multiple possibilities, and patients could activate different neural pathways. Regarding the Oddball test, no statistical significance was found, but, clinically, there was an improvement in the reaction time and the accuracy percentage in the control group compared to the experimental group. However, according to the MEC, the global cognitive abilities increased in the participants who were subjected to activities with IT. The contradiction between the results of the Oddball test and MEC, even without being significant in the Oddball test, could be because the patients tended to automate the routine. A global improvement at the cognitive level would lead the subject to deeply analyze each issue involved in the tasks (the sequence of the target stimuli, the time between stimuli, and the sequence of keys); consequently, more time would be lost when performing the tasks, and the accuracy (scores) of the routine would be decreased.

Everyday activities involve attention, efficient motor planning processes, and the useful inhibition of details that are unsuitable and irrelevant [39]. For all of this, it is necessary that the balance, gait, memory, and attention, among other functions, are in the best condition. Scherder et al. [40] recommended the unification of physical activity and cognitive rehabilitation strategies to increase functionality and autonomy. Interventions based on IT and feedback, such as those with the Nintendo^®^ Wii, improve physical–cognitive capacities; therefore, autonomy is optimized, as our results corroborate. In addition, the use of different types of feedback in such tasks might improve neural connections and compensate for possible deficiencies [17]. In this study, numerous types of feedback were used, with all of them being extrinsic because they came from external devices. These included a combination of acoustic and visual feedback. Although visual feedback is considered easier than acoustic feedback, the combination of both of them would further facilitate information processing [41], which is especially suitable for Alzheimer’s disease. Thus, in this study, visual information was considered essential and was easily followed by the patients. Acoustic feedback was used to reinforce the visual information and even as a motivating agent, e.g., by using stimulating sounds after the completion of a challenge.

Simultaneous, immediate, and terminal feedback were also employed. In addition, the KR (total score) and KP, depending on the game, were provided at the end of the task (game). The KP was limited in order avoid saturating the patients with information (e.g., the run time). In this study, the usual KP from the therapist was not provided to avoid bias in the results. Likewise, descriptive and prescriptive feedback for patients, which are often applied during therapeutic processes in clinical settings, were avoided.

According to other studies [17,42,43], combinations of feedback are frequently employed due to their effectiveness. In fact, the combination of concurrent, immediate, and visual feedback is one of the most common, followed by the combination of terminal, delayed, and acoustic feedback [17]. Although delayed feedback is considered particularly useful because of its long-lasting effect, in this study, it was omitted because it is more difficult for the user to process.

On the other hand, one of the great objectives in the gerontological sector is the prevention of falls. People suffering from dementia (with a fall incidence of 80%) have a doubled risk of falling in comparison with the healthy elderly population [20,44]. In addition, mobility problems are considered relevant risk factors for falls [45]. The values found in this study showed that the experimental patients had a reduced risk of falling in comparison with the control group, which is supported by other research [7,8,33]. In this sense, despite the small sample, which was proposed as a limitation, this study showed the benefits of physical–cognitive tasks by using feedback-based technology with therapeutic supervision for both the physical and cognitive capacities of the patients. This therapy also benefits from other advantages, such as its low cost, feasibility of use at home, and creation of intergenerational bonds.

Based on the above, in addition to proposing prospective studies similar to this one with a larger sample, we propose carrying out research to analyze the efficacy of the ANT in facilitating decisions on the ideal therapy for each case of Alzheimer’s disease. Thus, we suggest analyzing the different parameters of the tool (number of trials, duration of the stimulus, size of the visual stimulus, etc.) in detail and identifying the most appropriate combinations of parameters, i.e., protocols for action, in different neurodegenerative diseases. A final proposal would be to carry out studies of the efficacy of different combinations of feedback in cases of Alzheimer’s disease—for example, a similar trial (using physical–cognitive tasks with IT) in which visual and immediate feedback is applied in one group and visual, acoustic, and immediate feedback is applied in another.

## 5. Conclusions

This study concluded that there was an improvement in memory, balance, gait, and risk of falling in patients with Alzheimer’s disease who were subjected to the use of physical–cognitive tasks that involved feedback-based technology. However, the findings on attention were inconclusive. In addition, these could constitute an effective strategy for preventing the progression of the disease and improving neuroplasticity. The physical–cognitive tasks with IT included combinations of visual feedback, acoustic feedback, simultaneous feedback, immediate feedback, terminal feedback, knowledge of the results, and knowledge of performance; this was found to be suitable. The outcomes should be treated with caution as this was a pilot study.

Other benefits of this method are that it is an effective low-cost strategy that is framed in the patient’s own home, and it is an intergenerational approach.

Knowledge of the state of attentional networks by means of the Attention Network Test could be useful for selecting the ideal therapy in each case of Alzheimer’s disease.

## Figures and Tables

**Table 1:** Intervention protocol.

Day 1	Day 3	Day 8	Day 62	Day 65
Report about the study, signing of consent forms (legal guardian), and anamnesis.	Initial evaluation for each study (application of the tests).	Starting of treatment in the experimental group (first session).	Last treatment session in the experimental group.	Final evaluation for each study (re-test).
About 60 min. CG and EG.	About 60 min. CG and EG.	16 sessions in total (twice per week); 30 min. per session. EG.	EG	CG and EG.

Abbreviatures: CG: control group; EG: experimental group.

**Table 2:** Patient characteristics.

Patients	1	2	3	4	5	6	7	8	9	10	11	12	13	14	15
Group	EG	CG	CG	CG	EG	EG	CG	EG	CG	EG	CG	CG	EG	CG	EG
Genre	M	W	W	M	W	W	M	W	W	W	W	M	W	W	W
Subgroup	SS	SS	SS	MS	SS	MS	MS	MS	MS	SS	SS	MS	SS	SS	MS
Dominance	RH	RH	RH	RH	RH	RH	RH	RH	RH	RH	RH	RH	RH	RH	RH
Level of education	BS	BS	BS	SS	SS	PE	BS	PE	PE	BS	BS	PE	BS	BS	PE
Sleeping hours	11	9	11	9	8	9	9	8.5	9	7	9	9	8	10	9
Weight (kg)	54	73.5	71	70	80.5	79	80	57	69.6	62.4	83.2	103.4	60.7	69	56.6
Age	62	79	82	83	81	80	79	79	77	79	66	82	82	76	74
Other diseases	Pk DM	O	Pk	Sk DM	Arth	Sk DM	PD DM	Arth DM	Pk	Arth	Arth DM	Pk	O	Arth	Sk
Drug treatment for Alzheimer’s	Me	Me	D	R	D	R	R	R	R	D	Me	R	Me	Me	R

Abbreviatures: EG: experimental group, CG: control group; M: man, W: woman; SS: severe stage (4–5 points), MS: mild stage (3 points); RH: right-handed; BS: basic studies, PE: primary education; Pk: Parkinson (no other parkinsonian syndromes), Sk: stoke; DM: diabetes, Arth: arthrosis, O: osteoporosis, PD: pulmonary disease; R: Rivastigmine, Me: Memantine, D: Donepezil.

**Table 3:** Baseline features of the groups.

Dependent Variables (Scales and Test)	Statistical Tests	*p*
Age	Mann–Whitney U test	0.463
GDS	Student’s *s*-test	0.216
MEC	Student’s *s*-test	0.267
Berg scale	Mann–Whitney U test	0.397
Tinetti scale	Mann–Whitney U test	0.072
Timed up and Go Test	Mann–Whitney U test	0.867
Oddball(Accuracy)	Mann–Whitney U test	0.613
Oddball (Reaction time)	Mann–Whitney U test	0.189
ANT (Alert)	Welch’s *t*-test	0.059
ANT (Orienting)	Welch’s *t*-test	0.158
ANT (Executive)	Welch’s *t*-test	0.970

Abbreviations: GDS: Geriatric Deterioration Scale; MEC: Cognitive Mini-Examination; ANT: Attention Network Test.

**Table 4:** Descriptive variables, contrast test, interaction test between groups, and effect size.

Dependent Variables (Scales, etc.)	Groups	Mean-SD or Median-Interquartile Range (Indicated by *)	Contrast Test (*p* < 0.05)	Interaction between Groups (*p* < 0.05)	Effect Size
GDS (test)	CG	4.25 ± 1.03			
	EG	3.50 ± 1.04		0.347	0.68
GDS (re-test)	CG	4.38 ± 1.30	0.598		
	EG	3.83 ± 1.32	0.172		
MEC (test)	CG	16.50 ± 5.29			
	EG	19.17 ± 4.70		0.029	0.14
MEC (re-test)	CG	15.38 ± 5.31	0.026		
	EG	19.17 ± 7.02	0.675		
Berg Scale (test)	CG	43 ± 8.5			
	EG	47.14 ± 6.09		0.105	0.76
Berg Scale (re-test)	CG	34 ± 10.43	0.070		
	EG	49.47 ± 5.99	0.248		
Tinetti (test)	CG	22.13 ± 2.74			
	EG	26 ± 4 *		0.029	0.68
Tinetti (re-test)	CG	19 ± 3.92	0.010		
	EG	28 ± 2 *	0.068		
Timed Up and Go (test)	CG	15.42 ± 17.76 *			
	EG	14.73 ± 2.94		0.141	0.65
Timed Up and Go (re-test)	CG	20.39 ± 6.38	0.401		
	EG	12.69 ± 2.73	0.020		
Oddball—Accuracy (test)	CG	83.25 ± 20.87 *			
	EG	85.75 ± 7.85		0.159	0.60
Oddball—Accuracy (re-test)	CG	84.50 ± 11.15	0.401		
	EG	84.75 ± 8.51	0.837		
Oddball—Reaction Time (test)	CG	401.25 ± 149 *			
	EG	436.50 ± 111 *		0.242	0.41
Oddball—Reaction Time (re-test)	CG	395 ± 89.75 *	0.401		
	EG	402.32 ± 80.09	0.499		
ANT—alert (test)	CG	−85.60 ± 192.87			
	EG	49.66 ± 116.49		0.161	0.59
ANT—alert (re-test)	CG	−88.20 ± 67.67	0.726		
	EG	−55.66 ± 61.33	0.118		
ANT—orienting (test)	CG	90.60 ± 196.07			
	EG	−3.5 ± 72.85		0.231	0.48
ANT—orienting (re-test)	CG	116.60 ± 121.80	0.459		
	EG	−22.16 ± 136.16	0.777		
ANT—executive (test)	CG	85.20 ± 54.78			
	EG	61.66 ± 36.36		0.04	1.19
ANT—executive (re-test)	CG	106.80 ± 151.22	0.374		
	EG	−60.66 ± 120.93	0.077		

## Data Availability

Not applicable.

## Abbreviations

ANT	Attention Network Test
Arth	Arthrosis
BS	Basic Studies
CG	Control Group
D	Donepezil
DM	Diabetes
EG	Experimental Group
GDS	Geriatric Deterioration Scale
IT	Informational Technology
LH	Left-handed
M	Man
Me	Memantine
MEC	Cognitive Mini-Examination
MMSE	Mini-Mental State Examination
MS	Mild stage
O	Osteoporosis
PD	Pulmonary Disease
PE	Primary Education
Pk	Parkinson
R	Rivastigmine
RH	Right-handed
SE	Secondary Education
Sk	Stoke
SS	Severe stage
W	Woman
